# Parent post-traumatic growth after a child's critical illness

**DOI:** 10.3389/fped.2022.989053

**Published:** 2022-09-29

**Authors:** Lauren M. Yagiela, Camera M. Edgar, Felicity W. K. Harper, Kathleen L. Meert

**Affiliations:** ^1^Division of Critical Care, Department of Pediatrics, Children's Hospital of Michigan, Detroit, MI, United States; ^2^Department of Pediatrics, Central Michigan University, Mount Pleasant, MI, United States; ^3^Department of Oncology, Karmanos Cancer Institute, Wayne State University, Detroit, MI, United States

**Keywords:** critical care outcomes, parents, pediatric intensive care, follow-up, post-traumatic growth

## Abstract

**Objective:**

Post-traumatic growth is the experience of a positive change after a traumatic event. Our objective is to characterize the factors associated with post-traumatic growth in parents after a child's pediatric intensive care unit (PICU) admission.

**Study design:**

A cross-sectional survey study examining post-traumatic growth and select independent variables in parents 1 year after a child's ≥72 h PICU admission for an acute illness or injury. The study was completed in parents of children discharge alive from a tertiary care PICU from January 1, 2017 to December 31, 2017. A mixed-effects linear regression model was built to evaluate the association of post-traumatic stress, anxiety, depression, resiliency, family function, and child function with post-traumatic growth.

**Results:**

Eighty-two parents of 52 children discharged alive in 2017 completed the survey. Fifty-two percent were ≥35 years and 64.3% were mothers. Median age of their children was 2.8 years (IQR 0.5–11.3) with a median hospital stay of 12 Days (IQR 6–20). Moderate-to-high levels of post-traumatic growth occurred in 67.1% of parents. Increased hospital length of stay (β Coeff 0.85; *p* = 0.004, 95% CI 0.27, 1.43) and parent post-traumatic stress symptoms (β Coeff 1.04; *p* = 0.006, 95% CI 0.29, 1.78) were associated with increased post-traumatic growth, and increased parent depression symptoms (β Coeff −1.96; *p* = 0.015; 95% CI −3.54, −0.38) with decreased post-traumatic growth.

**Conclusion:**

Longer child hospital stays and increased parent post-traumatic stress symptoms were associated with increased post-traumatic growth, while increased depression was associated with less post-traumatic growth. The impact of future PICU parent psychosocial interventions on parents may be best assessed using a dual outcome focused on both reducing negative mental health symptoms while concurrently promoting skills to facilitate parent adaptation and post-traumatic growth.

## Introduction

A child's pediatric intensive care unit (PICU) admission can be traumatic for parents (1–11). The trauma parents experience can lead to poor mental health outcomes after a child's discharge, including post-traumatic stress disorder (PTSD), anxiety, and depression (1, 7–11). However, studies by Colville et al. (12) and Rodiguez-Rey et al. (13, 14) suggest some parents experience a positive change called post-traumatic growth after a child's PICU admission. Post-traumatic growth is a process whereby an individual experiences a positive psychological change after struggling with stressful event (15, 16). Tedeschi and Calhoun (15, 16) conceptualize that post-traumatic growth can occurs when a traumatic event is severe enough to challenge an individual's assumptive world. The distress that results can lead to emotional and cognitive processing, which in turn can contribute to sense making and benefit finding and ultimately, post-traumatic growth (15, 16). A recent systematic review by Picoraro et al. (17) on post-traumatic growth in pediatrics, focused mainly on parents and caregivers, posited that similar mechanisms lead to post-traumatic growth in parents and caregivers in pediatrics. Post-traumatic growth has been described as occurring in any of five areas including greater appreciation of life, improved interpersonal relationships, greater personal strength, recognition of new possibilities in one's life course, and spiritual or religious growth (15, 16). Rates of post-traumatic growth in parents after a child's PICU admission vary (12–14). In a study from Spain, 37% of parents had a medium-to-high level of post-traumatic growth, while in a UK study, 88% experienced a “great” or “very great” degree of positive growth in at least one area (12, 13). Factors that have been associated with parent post-traumatic growth after a PICU admission include higher perceived severity of illness, increased depression and anxiety, and presence of post-traumatic stress disorder (PTSD) (12–14).

While prior studies have examined rates of parent post-traumatic growth after a child's PICU admission in European populations, rates in a US population remain largely unknown. Additionally, current studies have explored parent post-traumatic growth at 4–6 months after a child's discharge but have yet to study this outcome longer-term after a child's discharge. Further, we lack robust information on how family functioning, parent resiliency, and post-discharge child functional status impact post-traumatic growth. Gaining a broader understanding of parent post-traumatic growth rates and examining how critical factors impact parent post-traumatic growth can inform PICU-based parent psychosocial interventions, such as outcomes interventions could target. As such, our study objectives are, in patients after a child's PICU admission of ≥72 h due to an acute illness or injury, to describe: (1) prevalence of parent post-traumatic growth; (2) child, parent, and family factors associated with parent post-traumatic growth.

## Materials and methods

### Design, setting, and participants

This was a cross-sectional survey study of post-traumatic growth in parents after a child's PICU admission in an urban tertiary care center. All parents of children < 18 years who met inclusion criteria were mailed, to the primary address for the child, a survey 10–14 months after a child's hospital discharge. The survey measured the primary outcome of post-traumatic growth and additional independent variables. If a survey was not returned, the parent was contacted *via* phone to complete the survey. Inclusion criteria were parents of children with ≥72 h PICU admission for an acute illness or injury who were discharged alive between January 1, 2017 and December 31, 2017. An acute illness included any unplanned PICU admission for diagnoses such as respiratory failure, sepsis, neurologic dysfunction, and trauma. A mother, father, or mother-father dyad of the same child could be recruited. Medical chart abstraction was used to collect the child's demographics, baseline health and hospitalization characteristics, and post-discharge outcomes. The study was approved by the Wayne State University and Detroit Medical Center Institutional Review Board (IRB #094717BE3, approved 10/24/2017). Parents received an information sheet by mail describing the study and indicated their consent by completing the survey.

### Primary outcome & independent variables

The primary outcome of post-traumatic growth was assessed with the Post-traumatic Growth Inventory (PTGI) developed by Tedeschi and Calhoun (16). The PTGI includes 21 items that assess five subscales: appreciation of life, interpersonal relationships, personal strength, new possibilities, and spiritual change. Respondents rate the degree to which they experienced each item using a Likert scale from “no change” (0) to “very great degree of change” (5). Total scores range from 0 to 105. Scores equal to or >63, an average score of “moderate change,” were considered consistent with the development of post-traumatic growth based on prior literature (13). The average subscale score was calculated by dividing the subscale total by the number of subscale items. The PTGI has demonstrated excellent internal consistency (Crohnbach's α = 0.90) (16).

Independent variables were selected by the research team by reviewing prior literature on parent post-traumatic growth in pediatrics. First, prior studies on parent post-traumatic growth have sought to control for and examine the associations between child factors including demographics, baseline health, and hospitalization characteristics and parent post-traumatic growth (12–14, 17–20). As such, the research team identified standard measures used in pediatric critical research to identify the independent variables for demographics, baseline health, and hospitalization characteristics (12–14, 21, 22). Next, parent independent variables were chosen by the research team based on review of prior literature on parent post-traumatic growth in pediatrics (12–14, 17–20). Specifically, we chose independent variables that underlie the mechanisms leading to parent post-traumatic growth in pediatrics described in a recent systematic review by Picoraro et al. and independent variables found to be associated with parent post-traumatic growth in pediatrics (12–14, 17–20).

Child independent variables included demographics (age, gender), baseline health (comorbid conditions, prior hospital admissions), hospitalization characteristics (primary admission diagnosis; PRISM-IV calculated severity of illness; hospital and PICU length of stay; adjunctive therapies including arterial and central lines, vasopressors, renal replacement therapy, and mechanical ventilation) and post-discharge outcomes (functional status, re-hospitalization at index hospital either on general care floor or PICU prior to survey completion). Functional status was assessed by parents on the survey using the Functional Status II (R) (FSIIR) (22). The FSIIR is composed of 14 items and has good internal consistency (Crohnbach's α >0.80). Scores range from 0 to 28, with higher scores indicative of better function.

Parent independent variables included demographics and parent-reported post-traumatic stress, anxiety, depression, resiliency, and family functioning. Demographics included age, gender, employment status, household income, education, and number of children the in home. Post-traumatic stress symptoms were assessed with the Short PTSD Rating Interview (SPRINT) (23, 24). The SPRINT is composed of 8 items and has good internal consistency (Crohnbach's α = 0.77). Scores range from 0 to 32 and a score of 14 or more has a 95% sensitivity to detect PTSD in populations with assumed PTSD rates of 20% (23, 24). Anxiety and depression were assessed with the Hospital Anxiety and Depression Scale (HADS) (25). The HADS includes 14 items and has good internal consistency [average Crohnbach's α = 0.83 (anxiety), 0.82 (depression)] (26). Subscale scores range from 0 to 21, with scores of 11 or more considered concerning for moderate to severe anxiety or depression (25). Resiliency was assessed with the Brief Resiliency Scale (BRS) (27, 28). The BRS contains six items and has good internal consistency (Crohnbach's α = 0.70–0.90). Scores range from 5 to 30. When the total score is divided by 6, a score 4.31 or greater is considered consistent with high resilience (29). Family function was assessed with the Family Assessment Device-General Functioning (FAD-GF) (30, 31). The FAD-GF contains 12 items and has good internal consistency (average Crohnbach's α = 0.78). Scores range from 12 to 48. When the total raw score is divided by 12, a score of two or greater indicates unhealthy family functioning.

### Statistical analysis

Data analysis was done using STATA version 14 (STATA, College Station, TX). Surveys underwent double entry and discrepancies were evaluated and corrected by the principal investigator. Participants were excluded from the final analysis if they were missing >20% of the items on the PTGI. All other survey measures were scored and included in the final analysis if ≥80% of the data was present. To calculate a survey measure score in the presence of missing data, missing item responses were replaced by the mean of the completed items. If two answers were selected for an item, the average value of the two answers was used. Categorical data are presented as frequencies with percentages and continuous data as median values with their interquartile range (IQR). We completed two separate analyses. First, descriptive statistics of the primary outcome of post-traumatic growth and independent variables were completed. Next, we built a mixed-effects linear regression model to evaluate the association of the independent variables with our primary outcome of post-traumatic growth. A mixed-effect linear model was used to account for the correlation between the responses of mother and father dyads. In the first model building step, an empty model was estimated to calculate the interclass correlation. Next, all independent variables were compared individually for their association with post-traumatic growth. Given prior research showing both a linear and curvilinear association between post-traumatic stress symptoms and post-traumatic growth (12, 13), post-traumatic stress symptoms alone and post-traumatic stress symptoms as quadratic were included. Candidate variables for the final model were those with a *p* value of < 0.2 and lacked collinearity (variance inflation factor < 2.5) with other candidate variables. The between-parent group variables (contextual effect) were excluded from the final model due to collinearity with between-parent variables. To build the final model, manual stepwise forward selection was performed until the addition of predictors failed to increase the amount of variance (Total *R*^2^) in post-traumatic growth scores explained by the model.

## Results

### Survey response & population characteristics

**Figure 1:**
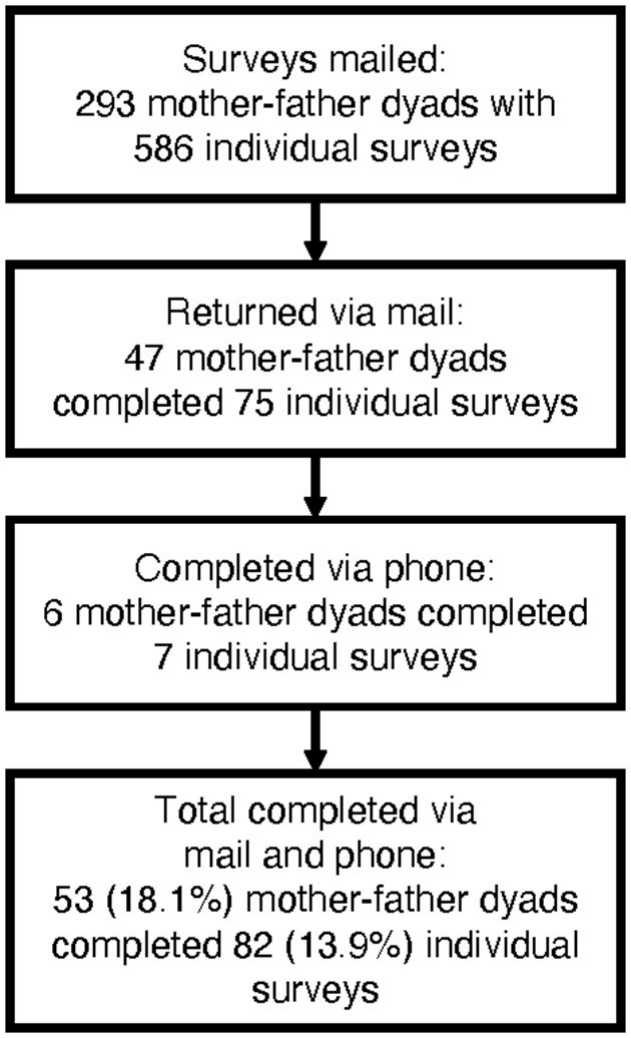
Recruitment flow diagram and survey response rate.

Eighty-two parents of 52 children discharged alive between January 1, 2017 and December 31, 2017 completed the survey. Eighteen percent of eligible families completed 13.9% of surveys mailed (Figure 1). The gender, age, and length of hospitalization of the children of parents who did and did not complete the survey were not statistically different (*p* = 0.56, *p* = 0.70, *p* = 0.70, respectively; Supplemental Table 1). The median time from discharge to survey completion time was 15 months (IQR 14–17). All participants completed at least 80% of the PTGI and < 1% of data points were missing (Supplemental Table 2). Fifty-one percent of parents were 35 years or older with a gender distribution of 64.6% mothers and 35.4% fathers (Table 1). Median age of their children was 2.8 years at hospital admission (IQR 0.5–11.3), 56.6% had a comorbid condition, and the lead primary diagnosis was respiratory conditions (Table 2). Median PICU length of stay was 6 days (IQR 4–12) and median hospital stay was 12 days (IQR 6–20). Fifty percent of the population were re-hospitalized prior to survey completion (Table 2).

**Table 1:** Parent demographics.

**Parent demographics**	***n* (%)**
**Age**, ***n*** **=** **82**	
18–24 years	6 (7.3)
25–34 years	34 (41.5)
35–44 years	24 (29.3)
>45 years	18 (21.9)
**Gender**, ***n*** **=** **82**	
Female	53 (64.6)
Male	29 (35.4)
**Employment status**, ***n*** **=** **82**	
Employed	50 (61.0)
Not employed or disabled	32 (39.0)
**Household yearly income**, ***n*** **=** **80**	
< $20,000	38 (47.5)
$20,000–$49,999	22 (27.5)
$50,000–$99,999	11 (13.8)
>$100,000	9 (11.2)
**Education**, ***n*** **=** **82**	
Some high school or high school degree	38 (46.3)
Some college or advance degree	44 (53.7)
**Children in home**, ***n*** **=** **81**	
1 child	22 (27.2)
2–3 children	37 (45.6)
4 or more children	22 (27.2)

**Table 2:** Child demographics, baseline health and hospitalization characteristics.

**Child demographics & baseline health (*n* = 53)**	**(*n* = 53)**
**Age at hospital admission, median (IQR)**	2.8 (0.5–11.3)
**Gender**, ***n*** **(%)**	
Female	20 (37.7)
Male	33 (62.3)
**Comorbid condition**, ***n*** **(%)**	30 (56.6)
Neurologic	18 (34.0)
Respiratory	15 (28.3)
GI	9 (17.0)
Genetic	8 (15.1)
**Prior hospital admission**, ***n*** **(%)**	33 (60.4)
Prior NICU hospitalization	16 (30.2)
Prior general ward hospitalization	25 (47.2)
Prior PICU hospitalization	20 (37.7)
**Child hospitalization characteristics**	**(*****n*** **=** **53)**
**Admission diagnoses**, ***n*** **(%)**	
Respiratory failure	26 (49.1)
Sepsis	7 (13.2)
Neurologic	6 (11.3)
Other	14 (26.4)
**PRISM-IV derived probability of death, median (IQR)**	3% (1–5%)
**Mechanical ventilation**, ***n*** **(%)**	
*Via* endotracheal tube	29 (54.7)
*Via* tracheostomy	4 (7.5)
**Duration of mechanical ventilation for those who received, days, median (IQR)**	5.0 (2.7–8.1)
**Adjunctive therapies**	
Use of vasoactive medications^a^, *n* (%)	9 (17.0)
Central line	9 (17.0)
Arterial line	9 (17.0)
ECMO	0 (0)
CRRT	2 (3.8)
**Length of stay, days, median (IQR)**	
PICU	6 (4–12)
Hospital	12 (6–20)
**Child outcomes**	**(*****n*** **=** **53)**
**Functional status (FSIIR), median (IQR)**	22 (18–24)
**Re-hospitalization**, ***n*** **(%)**	27 (50.9)
General ward	21 (40.4)
PICU	19 (35.9)

IQR, interquartile range; NICU, neonatal intensive care unit; PICU, pediatric intensive care unit; ECMO, extracorporeal membrane oxygenation; CRRT, continuous renal replacement therapy; PRISM-IV, pediatric risk of mortality version 4.

a Vasoactive medications include epinephrine, norepinephrine, vasopressin, dopamine, and milrinone.

### Parent survey outcomes

The median post-traumatic growth inventory score was 73 (IQR 50–84) with 67.1% of parents with scores consistent with moderate-to-high post-traumatic growth (Table 3). The subscales with the highest median growth included spiritual change (4, IQR 2–4.5) and appreciation of life (4, IQR 3.2–4.7) (Table 3). The survey outcomes for post-traumatic stress disorder, anxiety, depression, resiliency, and family functioning are described in Table 3.

**Table 3:** Parent survey outcomes.

**Parent survey outcomes *(n* = 82)**	**Median score (IQR)**	**Achieving threshold value**	
		**Description of threshold**	***n* (%)**
**Post-traumatic growth inventory** ***(n*** **=** **82)**	73 (50–84)	**Moderate-to-high post-traumatic growth**	55 (67.1)
**Subscales:**			
Relating to others	3.3 (2–4.1)		
New possibilities	3.0 (1.6–3.8)		
Personal strength	3.5 (2.8–4.3)		
Spiritual change	4.0 (2.0–4.5)		
Appreciation of life	4.0 (3.2–4.7)		
**Short PTSD rating scale (SPRINT)** ***(n*** **=** **83)**	11 (5–20)	**Concern for PTSD**	33 (40.7)
**Hospital anxiety and depression scale (HADS)** ***(n*** **=** **84)**			
Anxiety	7 (4–11)	**Moderate-to-high anxiety**	21 (25.6)
Depression	4 (1–7)	**Moderate-to-high depression**	11 (13.4)
**Brief resiliency scale** ***(n*** **=** **84)**	4.4 (3.8–5.0)	**High resiliency**	18 (22.0)
**Family assessment device** ***(n*** **=** **84)**	1.8 (1.3–2.1)	**Concern for unhealthy family functioning**	33 (40.2)

IQR, interquartile range.

### Independent variables associated with post-traumatic growth

The interclass correlation of the empty model was 62.5. In bivariate analyses, the child independent variables associated (*p* < 0.2) with post-traumatic growth included male gender (β coeff 12.16; *p* = 0.091; 95% CI −1.92, 26.24), hospital length of stay (β coeff 0.91; *p* = 0.003; 95% CI 0.31, 1.51), PICU length of stay (β coeff 0.74; *p* = 0.055; 95% CI −0.02, 1.49), and re-hospitalization prior to survey completion (β coeff −10.58; *p* = 0.130; 95% CI −24.28, 3.12) (Supplemental Table 2). The parent independent variables associated (*p* < 0.2) with post-traumatic growth included age ≥35 years (β coeff −9.07; *p* = 0.124; 95% CI −20.64, 2.50), male gender (β coeff −9.02; *p* = 0.025; 95% CI −16.89, −1.15), employment (β coeff −8.21; *p* = 0.101; 95% CI −18.02, 1.60), income >$20,000/year (β coeff −12.98; *p* = 0.032; 95% CI −24.85, −1.12), parent post-traumatic stress symptoms (β coeff 0.48; *p* = 0.137; 95% CI −0.15, 1.12), quadratic relationship of parent post-traumatic stress symptoms (β coeff 0.07; *p* = 0.039; 95% CI 0.000, 0.13), parent depression (β coeff −0.90; *p* = 0.184, 95% CI −2.23, 0.43), and family function (β coeff −6.14; *p* = 0.198; 95% CI −15.49, 3.20) (Supplemental Table 2).

In a mixed effect linear regression model, child length of stay in hospital (β coeff 0.85; *p* = 0.004, 95% CI 0.27, 1.43), parent post-traumatic stress symptoms (β coeff 1.04; *p* = 0.006, 95% CI 0.29, 1.78), and parent depression (β coeff −1.96; *p* = 0.015; 95% CI −3.54, −0.38) were significantly associated with post-traumatic growth scores (Total *R*^2^ = 0.259, Table 4). In the final model, the number of observations used were 78 and number of groups were 52. Parents with missing data in household income and post-traumatic stress symptoms were excluded by statistical software.

**Table 4:** Mixed effect linear regression model assessing for variables associated with post-traumatic growth.

***n* = 80**	**β co-efficient^c^**	**95% confidence interval**	***P*-value**
**Length of stay in hospital**	0.85	0.27, 1.43	0.004
**Gender**			
Female	Reference		
Male	−7.67	−16.36, 0.99	0.080
**Household income**			
< $20,000	Reference		
>$20,000	−10.1	−21.81, 1.59	0.090
**Employment**			
Unemployed	Reference		
Employed	2.83	−7.54, 13.21	0.592
**Parent post-traumatic stress symptoms**	1.04	0.29, 1.78	0.006
**Parent depression**	−1.96	−3.54, −0.38	0.015
**Parent assessed family function** ^b^	−6.95	−17.09, 3.19	0.179

a Vasoactive medications include epinephrine, norepinephrine, vasopressin, dopamine, and milrinone.

b Higher scores indicative of unhealthy family function.

c Unstandardized beta co-efficient.

PTSD, post-traumatic stress symptoms.

Total R^2^ = 0.259, number of observations = 78, number of groups = 52.

## Discussion

Our findings demonstrate that nearly 70% of parents in a US-based sample in an urban children's hospital experience post-traumatic growth after a child's unexpected PICU admission. This finding is consistent with ranges of 37% to 88% in similar studies in European populations (12–14). Similar to prior studies, we found that most post-traumatic growth occurred in spiritual change and appreciation of life (12–14). Interestingly, only 21% of parents noted high resiliency and 13–41% of parents reported unhealthy family functioning and poor mental health outcomes including post-traumatic stress symptoms, anxiety, and depression. In a mixed-effect linear regression model that accounted for the correlation between mother-father scores, a longer length of child hospital stay and increased parent post-traumatic stress symptoms were associated with increased post-traumatic growth and increased depression symptoms were associated with decreased post-traumatic growth. We failed to find expected relationships between other factors, such as resiliency and family functioning, with post-traumatic growth.

Our findings that longer length of stay was associated with increased post-traumatic growth and that increased depression symptoms were associated with decreased post-traumatic growth differs from prior findings on parent post-traumatic growth in pediatrics. Regarding hospital length of stay, a study of parents of children with chronic illness found that a shorter length of stay was associated with increased post-traumatic growth (18) and a study of parents after a PICU admission found no association (12). A possible mechanism for our findings is that parents with longer hospital stays have a greater opportunity to develop a therapeutic alliance with PICU staff. Such an alliance would foster mutual understanding, caring, and trust to exist between provider and parent (32), thereby potentially fostering the cognitive processing that leads to post-traumatic growth. Regarding depression, prior studies have found either a positive association between depression and post-traumatic growth (13) or no relationship (12). The difference between our finding and prior studies could be due to different timing of outcome measurement. Prior studies measured these variables 6 months after a child's PICU discharge. However, our study measured these variables 1 year after a child's discharge, at which point longstanding depression symptoms could be interfering with a parent's ability to perceive post-traumatic growth.

In contrast, our finding of a positive relationship between post-traumatic growth and post-traumatic stress symptoms is similar to prior pediatric studies. Studies of parents of pediatric patients in the PICU, neonatal intensive care unit, and after surgery for a congenital defect have found a positive relationship between post-traumatic growth and post-traumatic stress symptoms (13, 19, 20). Another study in PICU parents found a curvilinear relationship, noting that the most post-traumatic growth occurred in parents with moderate post-traumatic stress symptoms rather than those with low or high symptoms (12). These finding suggest that some degree of post-traumatic stress symptoms provides the richest context for prompting the occurrence of post-traumatic growth.

However, we failed to find that parent resiliency and family function were significantly associated with post-traumatic growth. Resiliency has been shown to have a direct effect on post-traumatic growth in parents after a child's surgery for congenital anomalies (20). However, in a study of parents after their child's PICU admission, resiliency was not directly associated with post-traumatic growth but a path analysis revealed that resiliency was associated with increased positive emotions and positive emotions were in turn associated with post-traumatic growth (14). This suggests that resiliency could play a role in PICU parent post-traumatic growth through mediators like emotions and mental health. Regarding family functioning, we also failed to find an association between family functioning and post-traumatic growth, which has been found in parents of children after cancer (18).

Our study findings should be interpreted in light of some potential limitations. First, this was a single-center cross-sectional study. With a cross-sectional study, we lack data on parent baseline mental health prior to their child's PICU admission. As such, the mental health symptoms measured at 1 year after a child's PICU admission could reflect mental health symptoms that predate the child's PICU admission or mental health symptoms due to the child's PICU admission or a combination of both. However, regardless of the cause of these mental health symptoms, we found associations between mental health symptoms and post-traumatic growth. Additionally, we had a lower than expected survey response rate. While we have data noting no difference in the age, gender, and length of hospital stay of the children of parents who did and did not participate, we lack data comparing parent metrics of those who did and did not participate. Further, while surveys were mailed to the primary address for the child, data on whether mother-father dyads lived in the same household was not collected, which could have affected family functioning scores. While these factors could limit the generalizability of our findings, the data presented here furthers our understanding of post-traumatic growth, including how critical family, parent, and child factors could impact the level of parent post-traumatic growth.

Finally, our study findings and prior literature suggest two key directions for future research on parent outcomes after a child's PICU admission, including post-traumatic growth. First, future research on parent post-traumatic growth could include a focus on standardizing study protocols to improve the ability to combine results from different studies and interpret parent outcome results (33–35). This could include standardizing across various studies the study inclusion criteria, the instruments used to measure parent mental health and parent post-traumatic growth, and the timing of outcome data collection (33–35). Second, psychosocial interventions for PICU parents could feasibly target a dual parent outcome (12–14, 17–19). Currently, most interventions seek only to reduce poor mental health outcomes, like post-traumatic stress symptoms, anxiety, and depression (11, 36–39). Building on this work, a dual outcome could focus on both reducing negative mental health sequelae and promoting outcomes and skills that facilitate adaptation during future adverse experiences. Our study and prior literature support the feasibility of a dual outcome. These data demonstrate that parents can experience post-traumatic growth after a child's severe illness and that post-traumatic growth can co-occur with post-traumatic stress symptoms, depression, and anxiety (12–14, 17–19). One such future adverse experience that PICU parents could experience is their child being re-hospitalized. In the 2 years after a PICU admission, up to 50% of children will be re-hospitalized and up to 70% of these hospitalizations are in the PICU (40, 41). Targeting a dual outcome could help better prepare parents for the next hospitalization.

## Conclusion

Our findings demonstrate that a year after a child's unexpected PICU admission nearly 70% of parents experience post-traumatic growth. Longer child hospital stays and increased parent post-traumatic stress symptoms were associated with increased post-traumatic growth, while increased depression was associated with less post-traumatic growth. The impact of future PICU parent psychosocial interventions on parents may be best assessed using a dual outcome focused on both reducing negative mental health symptoms while concurrently promoting skills to facilitate parent adaptation and post-traumatic growth.

## Data availability statement

The raw data supporting the conclusions of this article will be made available by the authors, without undue reservation.

## Ethics statement

The studies involving human participants were reviewed and approved by Wayne State University IRB. Written informed consent for participation was not required for this study in accordance with the national legislation and the institutional requirements.

## Author contributions

LY conceptualized and designed the study, carried out data analysis, drafted the manuscript, and reviewed and revised the manuscript. CE assisted with survey administration and data collection. FH and KM assisted in designing the study and reviewed and revised the manuscript. All authors approved the final manuscript as submitted and agree to be accountable for all aspects of work.

## Funding

This work was funded by Ashok and Ingrid Sarnaik Endowment for Junior Faculty Fund Grant.

## Conflict of interest

The authors declare that the research was conducted in the absence of any commercial or financial relationships that could be construed as a potential conflict of interest.

## Publisher's note

All claims expressed in this article are solely those of the authors and do not necessarily represent those of their affiliated organizations, or those of the publisher, the editors and the reviewers. Any product that may be evaluated in this article, or claim that may be made by its manufacturer, is not guaranteed or endorsed by the publisher.
