# Supplementary material for: Parent post-traumatic growth after a child's critical illness
Source: Front Pediatr. 2022 Sep 29;10:989053. doi: 10.3389/fped.2022.989053 (PMC9557288; doi:10.3389/fped.2022.989053)
Supplement: Supplementary file 1 [file Table_1.DOCX]

**Supplemental Data Table 1: Child demographic and hospital length of stay of children of parents that did and did not complete the study survey**

| **Child Demographics &**  **Hospital Length of Stay** | **Children of parents who did complete survey**  **(n=53)** | **Children of parents who did not complete survey**  **(n=240)** | **P value** |
| --- | --- | --- | --- |
| Age at hospital admission, y, median (IQR) | 2.8 (0.5-11.3) | 2.5 (0.5-8.9) | 0.695 |
| Gender, *n* (%)  Female  Male | 20 (37.7)  33 (62.3) | 101 (42.1)  139 (57.9) | 0.561 |
| Length of Hospital stay, d, median (IQR) | 12 (6-20) | 11 (7-19) | 0.708 |

IQR=interquartile range, y=years, d=days
