# Supplementary material for: Parent post-traumatic growth after a child's critical illness
Source: Front Pediatr. 2022 Sep 29;10:989053. doi: 10.3389/fped.2022.989053 (PMC9557288; doi:10.3389/fped.2022.989053)
Supplement: Supplementary file 2 [file Table_2.DOCX]

|  | Total missing data points,  *n/(number of items x 82 participants), (%)* | Participants with <80% of data items complete, *n* (%) |
| --- | --- | --- |
| All Survey Measures  79 items | 46/6478 (0.7) | 0 (0) |
| Demographics  5 items | 4/410 (1.0) | 0 (0) |
| Posttraumatic Growth Inventory  21 items | 9/1722 (0.5) | 0 (0) |
| Brief Resiliency Scale  5 items | 1/410 (0.2) | 0 (0) |
| Short PTSD Rating Interview  8 items | 8/656 (1.2) | 1 (1.2) |
| Hospital Anxiety and Depression Scale, n (%)  14 items | 4/1148 (0.3) | 0 (0) |
| Family Assessment Device - General Functioning Scale  12 items | 8/984 (0.8) | 0 (0) |
| Functional Status II (R)  14 items | 12/1148 (1.0) | 0 (0) |

**Supplemental Data Table 2: Missing Data**
