# Supplementary material for: Parent post-traumatic growth after a child's critical illness
Source: Front Pediatr. 2022 Sep 29;10:989053. doi: 10.3389/fped.2022.989053 (PMC9557288; doi:10.3389/fped.2022.989053)
Supplement: Supplementary file 3 [file Table_3.DOCX]

| **Supplemental Table 3:** Bivariate Analysis of Association Between Child and Parent Independent Variables with Posttruamatic Growth | | | |
| --- | --- | --- | --- |
| **Child Demographics & Baseline Health** | ***B* Coefficient^c^** | **95% Confidence Interval** | **P Value** |
| Age at hospital admission | -0.03 | -0.13, 0.07 | 0.604 |
| Gender  Female  Male | Reference  12.16 | -1.92, 26.24 | 0.091 |
| Comorbid Condition | -2.07 | -16.16, 12.03 | 0.774 |
| Prior hospital admission  Prior NICU hospitalization  Prior general ward hospitalization  Prior PICU hospitalization | 6.05  1.92  2.02  -7.60 | -8.16, 20.27  -13.25, 17.09  -11.97, 16.02  -21.89, 6.69 | 0.404  0.804  0.777  0.297 |
| **Child Hospitalization Characteristics** | ***B* Coefficient^c^** | **95% Confidence Interval** | **P Value** |
| Admission Diagnoses  Respiratory Failure  Sepsis  Neurologic  Other | Reference  -2.12  -1.97  -2.38 | -22.19, 17.94  -25.26, 21.32  -20.5, 15.8 | 0.835  0.868  0.797 |
| PRISM-IV derived probability of death | 63.66 | -144.42, 271.74 | 0.549 |
| Mechanical ventilation | 2.16 | -12.33, 16.64 | 0.770 |
| Duration of mechanical ventilation for those who received, d | 0.79 | -0.46, 2.03 | 0.216 |
| Adjunctive therapies  Use of vasoactive medications^a^  Central line  Arterial line  CRRT | -11.62  0.50  6.61  15.69 | -20.96, 6.57  -17.97, 18.97  -11.78, 24.99  -20.96, 52.34 | 0.211  0.957  0.481  0.401 |
| Length of stay, d  PICU  Hospital | 0.74  0.91 | -0.02, 1.49  0.31, 1.51 | 0.055  0.003 |
| **Child Outcomes** | ***B* Coefficient^c^** | **95% Confidence Interval** | **P Value** |
| Functional Status (FSIIR) | 0.37 | -0.93, 1.68 | 0.574 |
| Re-hospitalization  General ward  PICU | -10.58  -8.26  -2.53 | -24.28, 3.12  -22.29, 5.78  -17.17, 12.11 | 0.130  0.249  0.735 |
| **Parent Demographics** | ***B* Coefficient^c^** | **95% Confidence Interval** | **P Value** |
| Age  <35 years  ≥35 years | Reference  -9.07 | -20.64, 2.50 | 0.124 |
| Gender  Female  Male | Reference  -9.02 | -16.89, -1.15 | 0.025 |
| Employment status  Not employed or disabled  Employed | Reference  -8.21 | -18.02, 1.60 | 0.101 |
| Household Income  < $20,000/year  > $20,000 /year | Reference  -12.98 | -24.85, -1.12 | 0.032 |
| Education  Some high school or high school degree  Some college or advanced degree | Reference  -1.21 | -13.59, 11.16 | 0.848 |
| Children in home, *n* (%)  1-3 children  >4 children | Reference  4.07 | -11.49, 19.62 | 0.608 |
| **Parent Survey Measures** | ***B* Coefficient^c^** | **95% Confidence Interval** | **P Value** |
| Parent PTSS  Parent PTSS x PTSS | 0.48  0.07 | -0.15, 1.12  0.000, 0.13 | 0.137  0.039 |
| Parent anxiety | 0.38 | -0.71, 1.47 | 0.496 |
| Parent depression | -0.90 | -2.23, 0.43 | 0.184 |
| Parent assessed family function^b^ | -6.14 | -15.49, 3.20 | 0.198 |
| Parent resiliency | 2.96 | -3.4, 9.32 | 0.362 |

^a^Vasoactive medications include epinephrine, norepinephrine, vasopressin, dopamine, and milrinone

^b^Higher scores indicative of unhealthy family function

^c^Unstandardized beta coefficient

PTSS = posttraumatic stress symptoms

d=days
